# On Cold Bathing in Typhoid Fever

**Published:** 1871-08

**Authors:** 


### On Cold Bathing in Typhoid Fever.

By DR. FEHRSEN.

(The Lancet, December 31st, 1870.)

Dr. Fehrsen, dating from Dresden, states that he has recently visited the military hospitals, and seen the numerous cases of typhoid fever brought from the seat of war in France. The type now raging among the French and Prussian troops is the typhus abdominalis of the Germans, or what is here known as typhoid or intestinal fever.

The fever cases are indiscriminately mixed with the wounded, and those suffering from dysentery. Many of these are removed to Dresden, and it was determined to try cold bathing. The mode adopted was, that as soon as possible after admission into the hospital, the temperature of the patient was ascertained. If the thermometer showed 104° Fahr., he was put for fifteen minutes into a bath of 59° Fahr., up to the neck; in cases of much headache or delirium, an additional quantity of cold water was poured over the head, or cold compresses were applied. A measurement, threequarters of an hour afterwards, uniformly showed a fall of two or three degrees. The cold bath was repeated during the first week from four to six times a day, or as often as the temperature attained to 102° or 103° Fahr., experience having shown that the rapid cooling down of the febrile heat to a normal temperature is a powerful means of mitigating the symptoms, shortening the duration of the disease, and favoring an early convalescene, besides obviating the necessity of prolonged bathing at a later period of the disease.

The most striking benefits derived from this cold bathing are—firstly, that the delirium is generally mild or easily subdued; secondly, an earlier return of sleep; thirdly, a total absence of bed-sores; fourthly, a less prostrate state of the system when the patient leaves the hospital. Women, being more manageable, bathe more willingly than men. Many cry or give way to moaning for a few minutes, until, by a little courage and lying quite still, the body gets accustomed to the cold. If the water, however, be in any way disturbed, immediately they commence moaning and crying, showing, probably, that the stratum of water in immediate contact with the body must have acquired a higher temperature from the evolution of the febrile heat. After the first bath the sick show less objection to its repetition, and some even like it. Dr. Fehrsen saw about two hundred cases thus treated. Nothing contraindicates the use of the bath except very feeble action of the heart, hemorrhages, or perforation of the bowels, nor is a little bronchitis considered as an obstacle. The lightest food, with very little claret, is all that is given. Quinine is administered for the express purpose of lowering the febrile heat, sixteen grains being given in two divided doses in the evening. The statistics of the Stadt-Krankenhaus, at the present time, contrast most favorably with those of a few years ago, when the mortality averaged 11¾ per cent., as at present it does not exceed 4 per cent.—*Half-Yearly Abstract.*

